# Update on the Integrated Nutrition Pathway for Acute Care (INPAC): post implementation tailoring and toolkit to support practice improvements

**DOI:** 10.1186/s12937-017-0310-1

**Published:** 2018-01-05

**Authors:** Heather Keller, Celia Laur, Marlis Atkins, Paule Bernier, Donna Butterworth, Bridget Davidson, Brenda Hotson, Roseann Nasser, Manon Laporte, Chelsa Marcell, Sumantra Ray, Jack Bell

**Affiliations:** 1 Schlegel-University of Waterloo Research Institute for Aging; Department of Kinesiology, University of Waterloo, Waterloo, ON Canada; 2 School of Public Health and Health Systems, University of Waterloo, Waterloo, ON Canada; 3 Alberta Health Services, Edmonton, AB Canada; 4 Ordre professionnel des diététistes du Québec, Montreal, QC Canada; 5 Concordia Hospital, Winnipeg, MB Canada; 6 Canadian Nutrition Society, Ottawa, ON Canada; 7 Winnipeg Regional Health Authority, Winnipeg, MB Canada; 8 Saskatchewan Health Authority, Regina, SK Canada; 9 Réseau de santé Vitalité Health Network, Campbellton Regional Hospital, Campbellton, NB Canada; 10 NNEdPro Global Centre for Nutrition and Health (Affiliated with: Cambridge University Health Partners, Wolfson College Cambridge and the British Dietetic Association), St John’s Innovation Centre, Cowley Road, Cambridge, UK; 11 School of Human Movement and Nutrition Sciences, The University of Queensland &, The Prince Charles Hospital, Brisbane, Australia

**Keywords:** Malnutrition, Nutrition screening, Subjective global assessment, Hospital, Evidence care pathway, Toolkit

## Abstract

The Integrated Nutrition Pathway for Acute Care (INPAC) is an evidence and consensus based pathway developed to guide health care professionals in the prevention, detection, and treatment of malnutrition in medical and surgical patients. From 2015 to 2017, the More-2-Eat implementation project (M2E) used a participatory action research approach to determine the feasibility, and evaluate the implementation of INPAC in 5 hospital units across Canada. Based on the findings of M2E and consensus with M2E stakeholders, updates have been made to INPAC to enhance feasibility in Canadian hospitals. The learnings from M2E have been converted into an online toolkit that outlines how to implement the key steps within INPAC. The aim of this short report is to highlight the updated version of INPAC, and introduce the implementation toolkit that was used to support practice improvements towards this standard.

**Electronic supplementary material:**

The online version of this article (10.1186/s12937-017-0310-1) contains supplementary material, which is available to authorized users.

The Integrated Nutrition Pathway for Acute Care (INPAC) published in 2015 was based on evidence and expert consensus. It was designed to be a feasible pathway and standard of nutrition care for health professionals to follow to improve the prevention, detection, and treatment of malnutrition and thus influence clinical care and outcomes [1]. The algorithm includes: nutrition screening with the Canadian Nutrition Screening Tool (CNST) [2]; classification of nutritional status using the Subjective Global Assessment (SGA) [3] for patients identified to be at risk by the screening tool; implementation of care as either standard (i.e. removing barriers to intake), advanced (i.e., typical diet interventions to promote intake), or specialized (i.e., comprehensive dietitian assessment and individualized treatments) nutrition care strategies; implementation of clinical monitoring including food intake monitoring; and nutrition care incorporated into the discharge process. Although designed with feasibility as a core principle, for actual uptake and use in professional practice as a standard, INPAC needed to be viewed as acceptable, practical, and easy to integrate into the current Canadian hospital context [4]. Further, understanding of how much tailoring was necessary to implement INPAC in diverse environments was required. This paper describes how INPAC was tailored in five diverse sites tasked with implementing INPAC during a one-year period and the resulting implementation toolkit that supports implementation by other hospitals. Results on success with implementation of INPAC with respect to uptake of practice activities, changes in clinical care, and patient outcomes, as well as continued use of INPAC and how to make these improvements, are presented elsewhere [5–8].

## The More-2-Eat project

More-2-Eat Phase 1 (M2E) was a participatory action research project designed to test and evaluate the implementation of INPAC in five diverse Canadian medical units [9]. Several theories and frameworks, including quality improvement were used to effect change in the hospital sites [9]. One objective was to determine if tailoring of the pathway was required to integrate this standard into the routine of care and meet the nutrition care needs of the patients included in the study. Data were collected in a variety of ways about the implementation process and sustainability of implemented INPAC activities [5–7, 10]. Based on the tailoring conducted by these five sites and discussion with M2E stakeholders, the algorithm was updated.

## Reaching agreement on INPAC changes

During the year of implementing INPAC (Jan-Dec 2016), key stakeholders from each site (M2E champions, and research associates) were asked by e-mail and at monthly calls to reflect upon their experience with INPAC and whether changes were required to refine the pathway. In December 2016, all M2E stakeholders attended a meeting to discuss project outcomes, including potential updates to INPAC. At the end of M2E Phase 1 (April 2017), the ideas suggested during the year were revisited by the key stakeholders to determine relevance and need for global changes to the algorithm, recognizing that context-specific tailoring would always be required with adoption of INPAC in a specific setting [11, 12]. Suggested changes were made by the M2E core team to the original documents (Algorithm, Guidance Document, and Instructions) and sent to all M2E stakeholders (M2E project collaborators, co-investigators etc.), and those involved in the original development of INPAC, for comment. The updated version was then resent to all authors for final approval. This process of tailoring, testing out, and confirming changes is consistent with knowledge translation frameworks for the maturation and adoption of an innovation [11, 12].

## INPAC updates

**Fig. 1:**
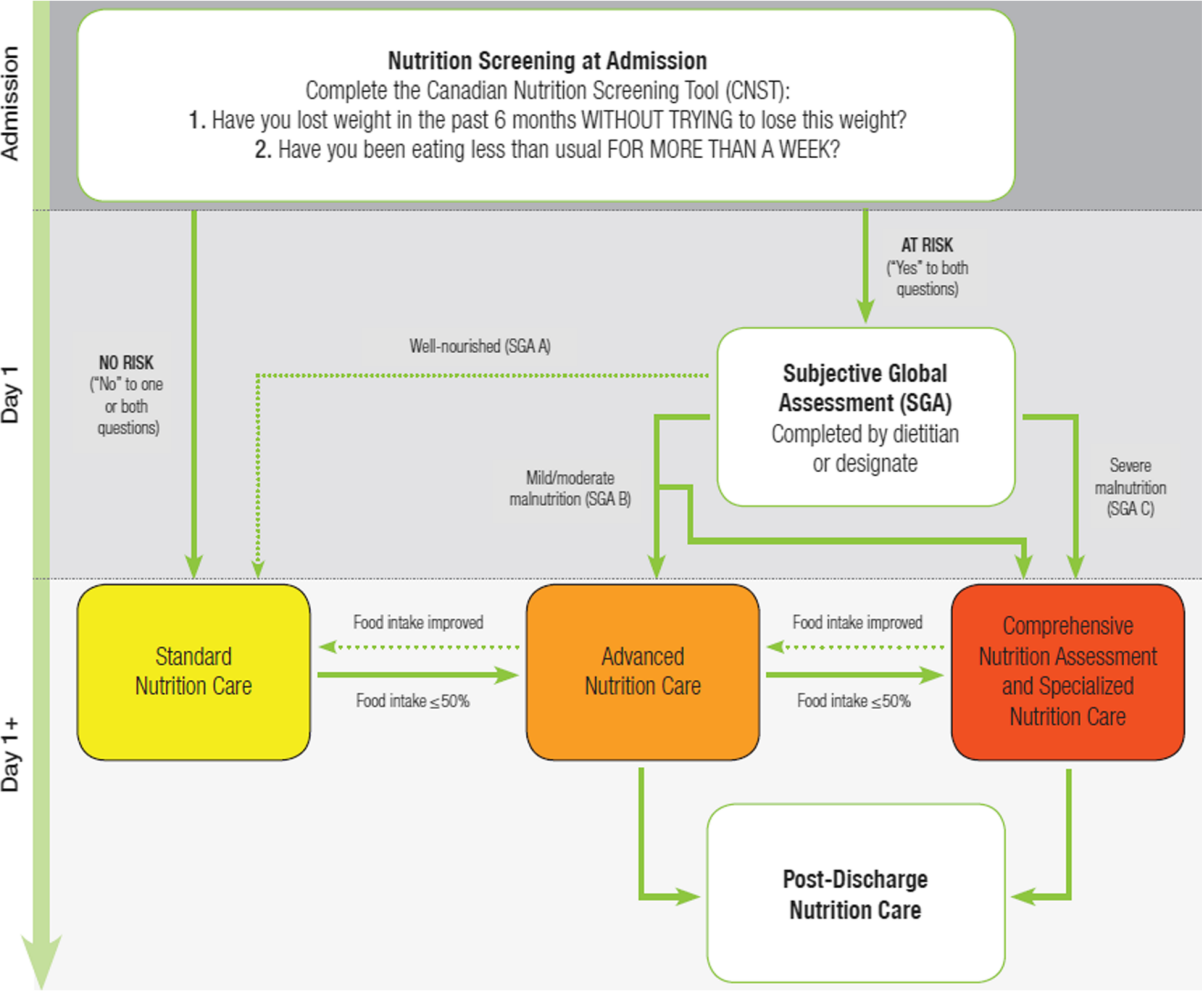
Updated version of the Integrated Nutrition Pathway for Acute Care

The updated version of the INPAC algorithm is shown in Fig. 1, with the revised Guidance Document (“What is INPAC? How Does It Work?”) and Instructions available as Additional files 1 and 2. Modifications are targeted at making INPAC adaptable and less prescriptive, and therefore more practical to accommodate the various hospital healthcare delivery systems and patient populations that may be interested in using INPAC. Changes were also made to clarify INPAC activities and enhance readability [12, 13]. Key revisions include:

### Screening

INPAC no longer specifies that an admission nurse should conduct nutrition screening. The CNST is simple to use and does not require specialized training. This specification of an admission nurse was removed to recognize that a variety of staff can conduct nutrition screening, such as a diet clerk. The hospital/unit can decide the most appropriate staff role to complete screening.

### Naming the levels of care

Specialized Nutrition Care is now called “Comprehensive Nutrition Assessment and Specialized Nutrition Care.” This change was made to recognize that a detailed assessment is typically required for SGA C (severely malnourished) patients to identify root causes of malnutrition and potential individualized treatments, including the appropriateness for enteral or parenteral nutrition, or palliative care. Specialized care also includes more frequent and detailed monitoring, overseen by the dietitian or nutrition professional.

Originally, INPAC included three levels of care (Levels A, B and C), in line with the three levels of SGA (A - well nourished, B - mild/moderately malnourished, and C - severely malnourished). Stakeholders found that this detail was unnecessary and implied that SGA B patients should only receive Advanced Care Strategies and not a comprehensive nutrition assessment. Thus, the alphanumeric labels were removed and the three levels are now labeled simply as Standard Nutrition Care; Advanced Nutrition Care; and Comprehensive Nutrition Assessment and Specialized Nutrition Care.

### Triaging SGA B patients

During the implementation of INPAC in phase 1 of M2E, it was noted that some SGA B patients also required Comprehensive Nutrition Assessment and Specialized Nutrition Care to establish a treatment plan. An additional arrow has been added to allow for clinical judgment to decide if a patient who is SGA B requires Advanced or Comprehensive Nutrition Assessment and Specialized Nutrition Care.

### Movement between care categories

The initial version of INPAC suggested transitions from Standard to Advanced, and Advanced to Specialized Care, in the event that treatment was not working and intake was poor, specifically ≤50%. This cut-point was justified based on research demonstrating that this level of intake was associated with a longer length of stay when adjusting for other covariates [14]. Conversely, Specialized Care patients whose malnutrition was beginning to be resolved could be moved back to Advanced Care if they were recovering well, described as ‘food intake improved’. An additional arrow from Advanced Care to Standard Care has been added to the updated INPAC to be inclusive of all potential transitions among care categories, and continues to rely on ‘food intake improved’ as the mechanism for making this decision. No specification is made on the amount of food intake required to be an ‘improvement’. This keeps the algorithm flexible and adaptable to the clinical context, which may vary in clinical nutrition resources, and allows clinical judgment for making treatment decisions.

### Food intake monitoring

Food intake monitoring was confirmed by all M2E sites as a primary mechanism for determining success with nutrition treatment. Some M2E sites had meal portion estimations provided by nursing staff in place before the project commenced, with some already using and others switching to the scale of 0, 25%, 50%, 75% and 100%. The original INPAC indicated that intake <50% should stimulate intervention. However, using the scale above, this lead to confusion of when to initiate such treatment. To promote clarity and support assessment of intake, the INPAC update indicates that food intake *less than or equal to* 50% be used to demonstrate low intake that needs intervention. It should be acknowledged that the amount of food provided to patients should meet their nutritional requirements. It was also noted during M2E that although food intake monitoring was already a routine practice by unit staff for some of the hospitals, typically on nursing vital statistics sheets, specific actions to address poor intake were not taken, which resulted in a gap in care. M2E sites had to resolve two issues when implementing INPAC: 1) ensuring accuracy of intake estimations, and 2) promoting an intervention when intake met the definition of being low (i.e., ≤50%). When implementing INPAC these are key areas for education and training, as well as process mapping.

All sites and stakeholders confirmed that food monitoring strategies should be in place to record patient intake and to respond appropriately when intake is low. Identification and resolution of barriers to intake are a strategy used in each of the care categories, with Standard Care focused on the most common strategies (e.g. opening food packaging), whereas Advanced and Specialized Care may require a variety of assessments to fully understand barriers to intake. Originally, the pathway had specific recommendations for frequency of monitoring food intake for Standard Care and Advanced Care patients. At all M2E sites, frequency and mode of food intake monitoring was tailored to what was practical and feasible, considering the current routines of nursing and food service staff, and how food intake monitoring could occur more systematically. For example, in one site, food intake monitoring became an added routine for food service workers at every meal, using a patient white board in their room to document intake. In another site, the nursing vitals sheet that documented intake at meals was used and processes for referral when intake was low were put into place. As a result of these tested processes, the specification on frequency of food intake monitoring was removed from the updated INPAC, allowing individual sites to consider how best to implement this strategy to promote the nutrition of all patients. As well, the specific recommendation for moving a patient from Advanced Care to Specialized Care after 3 days of food intake at less than 50% was also removed. Clinical judgment was used in M2E sites to move a patient up in intensity of care, often based on determining why intake was consistently low and providing, in some cases, further Advanced Care strategies to resolve these issues. Of note, all M2E sites decided that monitoring of *all* patients and asking about poor intake were seen as good ways of making sure that *every* patient (at nutrition risk or not) could be identified in a timely manner if their food intake was poor.

### Weight monitoring

Within the Guidance Document and Instructions, more emphasis is now placed on the need for accurate and regular weights. Admission weight and weekly weights are considered a minimum for best practice. The hospital/unit can determine when during the week a weight should be measured and by whom.

All other aspects of INPAC were found to be feasible, including timing of screening and completion of SGA for diagnosis [8]. The Instructions were streamlined to capture the noted changes and eliminate repetition.

## INPAC implementation toolkit

The learnings from M2E have been summarized in an INPAC Implementation Toolkit, which is available within the Canadian Malnutrition Task Force website. The toolkit focuses on *what* to do, highlighting all areas of INPAC, and *how* to change practice. The “how” section includes direction about: Getting Ready, team Engagement and Buy-in, Adopting the Change, and Keeping it Going. Adopting the Change specifically includes role delineation and development of competence within the workforce to complete INPAC activities such as screening, food monitoring, and conduct of SGA. Auditing and feedback were key behaviour change techniques that effected change and are incorporated into this section of the toolkit as well as in the section Keeping it Going. Developing hospital level standards and policy are other ways described to sustain INPAC. An example INPAC audit is a key resource that is part of the extensive collection of tools and resources incorporated into the toolkit, which also included posters, forms, guidance documents, and templates, all of which are freely available for use and can be downloaded. The toolkit is available online: http://nutritioncareincanada.ca/inpac/inpac-toolkit, as well as in an electronic version: http://m2e.nutritioncareincanada.ca/.

## Discussion

The aim of INPAC is to provide a pathway for triaging so all patients receive appropriate nutrition care in hospital. INPAC is similar to the European Society for Clinical Nutrition and Metabolism (ESPEN) nutritional algorithm, in that both recommend nutrition screening, assessment, monitoring (food intake, body weight etc.), and use of advanced care strategies such as oral nutritional supplements [15]. INPAC differs from the ESPEN algorithm in the routes of care, and is less prescriptive, particularly regarding nutritional targets (for energy, protein, and micronutrients), and enteral/parenteral nutrition. The ESPEN algorithm focuses on the guidelines [15, 16] and can provide additional detail and guidance for nutrition professionals, while INPAC encourages all hospital staff to be involved in nutrition care for all patients.

An effective nutrition care pathway should improve patient outcomes. Unfortunately, many studies that examine the impact of nutrition support in hospital are of low quality [17], making it difficult to know which strategies are most effective to include in a nutrition care algorithm. For this reason, INPAC was based on the available evidence, expert consensus [1], and has now been updated after feasibility testing in real world settings in the M2E study. However, this and other algorithms need to further demonstrate their effectiveness with respect to patient reported and clinical outcomes. Although INPAC is designed for medical and surgical units, all M2E units were medical, thus additional adaptations may be required as INPAC is used in surgical and other units. While the M2E study measured the effects of INPAC implementation on length of stay, barriers to food intake, frailty status, quality of life, dietitian resource utilization, and more [9], it was a feasibility study and sites were unable to implement the full pathway in the year provided. More information is required to see the longer-term effects on patient outcomes when the full pathway is fully implemented and sustained.

## Conclusion

Based on the implementation experience of the M2E project, the original INPAC was found to be feasible with a few updates to improve acceptability and uptake. To facilitate the implementation of the INPAC in hospitals, the toolkit is designed for clinicians and provides basic techniques for implementation and resources to support improving nutrition care practices. The updated INPAC has enhanced flexibility, supporting the necessary site-level tailoring, and the toolkit provides the necessary guidance on how to adapt INPAC to any acute care setting. The M2E study has also demonstrated that implementation of INPAC can change staff knowledge, attitudes and behaviour with respect to nutrition care and be sustained [6, 8], further demonstrating the acceptability and feasibility of this pathway. It is anticipated that these findings will result in a policy shift with respect to nutrition care in hospital. Updating the INPAC to make it feasible in all contexts was a necessary step towards the broad uptake of this care innovation.

## Additional files

Additional file 1: Updated INPAC Algorithm and Guidance Document. (PDF 267 kb)

Additional file 2: Updated INPAC Instructions. (PDF 65 kb)

## Abbreviations

CNST: Canadian Nutrition Screening Tool
INPAC: Integrated Nutrition Pathway for Acute Care
M2E: More-2-Eat implementation project
SGA: Subjective Global Assessment
