# Supplementary material for: Update on the Integrated Nutrition Pathway for Acute Care (INPAC): post implementation tailoring and toolkit to support practice improvements
Source: Nutr J. 2018 Jan 5;17:2. doi: 10.1186/s12937-017-0310-1 (PMC5756381; doi:10.1186/s12937-017-0310-1)
Supplement: Supplementary file 1 — Updated INPAC Algorithm and Guidance Document. (PDF 267 kb) [file 12937_2017_310_MOESM1_ESM.pdf]

INPAC: INTEGRATED NUTRITION PATHWAY FOR ACUTE CARE

What is INPAC?

An evidence-based algorithm developed by Canadian clinicians and researchers to detect, monitor, and treat malnutrition in acute care patients.

INPAC is based on the **key principle** that **an integrated approach** – or involvement from the whole health care team – is **required** to treat malnutrition. INPAC is a **minimum standard**; institutions that provide care beyond this minimum should continue to practice at their higher quality standard.

It is recommended that each hospital establishes an interdisciplinary team to promote and sustain the nutrition culture change required to implement INPAC.

INPAC: Designed to support **nutrition health** and **care**

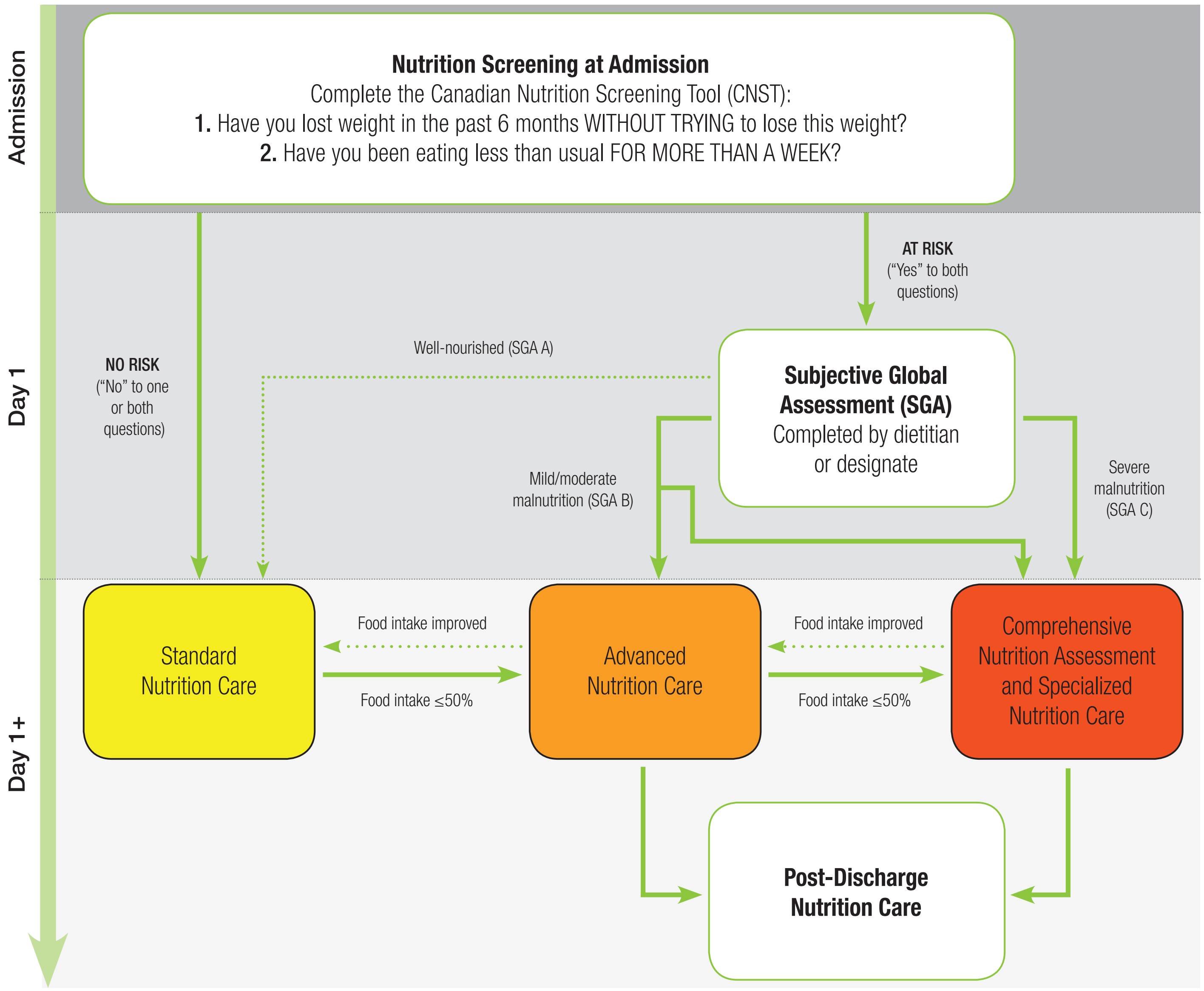

See reverse for further detail…

HOW DOES INPAC WORK?

INPAC involves nutrition **screening** – followed by a **subjective global assessment** in individuals deemed AT RISK – to **categorize patients** according to the **level of nutrition care** that they require: **Standard**, **Advanced**, or **Specialized**.

Nutrition Screening at Admission

If patient answers "Yes" to both Canadian Nutrition Screening Tool (CNST) questions listed on reverse side **OR** if any of the following apply to the patient:

- Requires enteral/parenteral nutrition
- Unable to complete CNST (e.g., language barrier, altered mental status)
- Transferred from critical care
- Has high nutrient requirement conditions (e.g., trauma, burns, pressure injuries, SIRS, etc.)

…then follow "**AT RISK**" pathway (on reverse).
If none of the above apply, then follow "**NO RISK**" pathway.

SIRS=systemic inflammatory response syndrome.

Subjective Global Assessment (SGA)

SGA is a gold standard for diagnosing malnutrition in hospitals. Dietitians or other trained professionals assess weight change, food intake, functional status, and body composition. SGA takes approximately 10 minutes.

Standard Nutrition Care

- Sit patient in chair or position upright in bed
- Ensure vision and dentition needs are addressed
- Address nausea, pain, constipation, diarrhea
- Confirm food is available between meals
- Ensure bedside table is cleared for tray set-up, open packages, provide assistance and encouragement to eat
- Encourage family to bring preferred foods from home
- Monitor and report key clinical observations/measurements:
    - Food intake
    - Duration of NPO/clear fluid intake
    - Hydration status
    - Body weight (preferably at admission and weekly)
    - Signs of dysphagia

NPO=nil per os (nothing by mouth).

Advanced Nutrition Care

Continue **Standard Nutrition Care** practices **AND**

- Assess and address barriers to food intake
- Promote intake with 1 or more of:
    - Nutrient dense diet (high in energy, protein, micronutrients)
    - Liberalized diet
    - Preferred foods
    - High energy/protein shakes/drinks (at/or between meals or as 'medpass', a small amount provided at each medication administration)

Comprehensive Nutrition Assessment and Specialized Nutrition Care

Continue **Standard & Advanced Nutrition Care** strategies where appropriate. Patient will undergo a comprehensive nutrition assessment completed by the dietitian, which involves:

- More detailed assessment of nutrition status using physical examination, body composition, food intake, clinical history, and biochemical markers
- Further identification of barriers to food intake (e.g., medication side effects, depression, etc.)
- Identification of eating behaviours that will support food intake
- Individualized treatment and monitoring
- Enteral and/or parenteral nutrition

Post-Discharge Nutrition Care

If patient is malnourished (SGA B or C) upon admission or during hospitalization, nutrition is an active issue in the discharge summary note (completed by dietitian, physician or nurse)

- Education provided to patient and family
- Referral to community resources (e.g., meal programs, grocery shopping)
- Send discharge summary with patient and a copy to family physician/care provider in the community; refer to appropriate resources in the community

Quality nutrition care and patient safety with INPAC

For more information and details on how to implement INPAC, please visit
http://nutritioncareincanada.ca/inpac/inpac-toolkit

This research was funded by the Canadian Frailty Network (CFN).

Food is Medicine

Canadian Malnutrition Task Force™ | le Groupe de travail canadien sur la malnutrition™

November 2017
