# Supplementary material for: Update on the Integrated Nutrition Pathway for Acute Care (INPAC): post implementation tailoring and toolkit to support practice improvements
Source: Nutr J. 2018 Jan 5;17:2. doi: 10.1186/s12937-017-0310-1 (PMC5756381; doi:10.1186/s12937-017-0310-1)
Supplement: Supplementary file 2 — Updated INPAC Instructions. (PDF 65 kb) [file 12937_2017_310_MOESM2_ESM.pdf]

Instructions for the Integrated Nutrition Pathway for Acute Care (INPAC)

The INPAC is...

- an evidence-based, field-tested algorithm for the prevention, detection, treatment, and monitoring of malnutrition
- designed for acute care medical and surgical patients
- designed to detect nutrition risk and malnutrition upon admission to hospital or as they develop during hospitalization, and not other conditions that require nutrition expertise
- a minimum standard for nutrition care; hospitals or units providing care above this minimum are encouraged to continue their high quality practice
- involves the entire healthcare team, and the patient and family in their nutrition care
- an algorithm for all team members which all have a role to play in improving nutrition care; roles are suggested but not exclusive to key professional groups.

To ensure successful implementation and sustainability, it is recommended that each hospital establish champions and an interdisciplinary team to implement the INPAC.

NUTRITION SCREENING AT ADMISSION

Within 24 hours of admission a designated professional completes the Canadian Nutrition Screening Tool (CNST):
1. Have you lost weight in the past 6 months WITHOUT TRYING to lose this weight?
2. Have you been eating less than usual FOR MORE THAN A WEEK?

- The Canadian Nutrition Screening Tool (CNST) is recommended as it is a valid, reliable, and quick tool to assess malnutrition risk.
- Nursing is often the first discipline to complete the patient admission assessment form and these two screening questions can be easily embedded into their current assessment tools.
- Others who interact with the patient within a few hours of admission (e.g. physician, diet technician) could also complete the nutrition screening.
- Page 2 of INPAC includes some situations where screening is not feasible (e.g. patient cannot answer questions) or where high risk factors/situations are present (e.g. patient requires nutrition support, severe cognitive barriers, transfer from intensive care etc.). In these instances, patients should be directly referred to a dietitian for nutritional evaluation and determination of nutrition care plan.
- Weekly re-screening is not necessary if monitoring for all patients routinely occurs (e.g. daily food intake, weekly weights).

Patients NOT AT nutrition risk: Standard Nutrition Care path

Standard Nutrition Care

- This is a standard of care provided to ALL patients.
- Standard Nutrition Care promotes food intake and weight monitoring of the patient so that poor food consumption can be identified and attended to early.
- Measure weight (at admission and weekly) and track food intake regularly.
- Health care aides, porters, dietary aides, and other personnel involved with meal service can complete food intake monitoring.
- Food intake of 50% or less at a meal suggests the need for closer follow up and determination of the need for more aggressive nutrition care strategies.
- Identify barriers to food intake (e.g. assistive devices needed to help with eating, difficulty self-feeding, swallowing assessment for dysphagia).
- Patient care teams are encouraged to collaborate to identify and address barriers, and to optimize intake for all patients e.g. minimizing interruptions during mealtime.
- Patients who are NPO or on clear fluids (continuously or intermittently) for > 3 days should be assessed using subjective global assessment (SGA) to determine their nutritional status.
- Page 2 of INPAC lists a variety of practices to support food intake.

Patients AT Nutrition Risk: Establish Malnutrition Diagnosis with Subjective Global Assessment

Subjective Global Assessment (SGA)

- Patients identified to be at nutrition risk require a diagnosis to confirm malnutrition. SGA is recommended for making a diagnosis and triaging further nutrition care.
- Dietitians or other trained professionals can conduct SGA.
- Ideally, SGA is completed within 24 hours of being screened 'at risk'. If the patient is admitted and screened at risk over the weekend, Advanced Nutrition Care procedures can be instituted until the SGA is completed.

Canadian Malnutrition Task Force™ | le Groupe de travail canadien sur la malnutritionMC
Advancing Nutrition Care in Canada / Améliorer les soins nutritionnels au Canada

- If patient is confirmed to be malnourished (SGA B or C) they should receive Advanced or Specialized Nutrition Care.
- Dietitians will decide, based on their clinical judgment, if a comprehensive nutrition assessment is required for SGA B patients.
- Dietitians will decide, based on their clinical judgment, if an SGA A patient requires Advanced Nutrition Care.

When a patient requires Advanced Nutrition Care

Advanced Nutrition Care

- Continue Standard Nutrition Care procedures, PLUS
- Provide more nutrient dense food to patients at and between meals to optimize oral intake.
- Methods to increase the nutrient density of the diet should be implemented at the next meal for these patients, such as:
  - o Provide high energy and high protein food offerings at and between meals;
  - o Small amounts of oral nutritional supplements (e.g. 60 mL) can be provided at each medication round;
  - o Providing preferred food;
  - o Have nutrient dense snacks available on the ward, and offer frequently.
- Assess barriers to food intake, for example, patient needs to have food packages opened, too many interruptions at meal times, etc.
- Collaboration with and education of the patient/family and health care team to improve intake is essential.
- Monitoring of food intake should be done daily.
- If overall food intake (meals, snacks, supplements) is 50% or less of what is provided, action may be required to address low intake. This action may include referral to a dietitian or nutrition support team to deliver Comprehensive Nutrition Assessment and Specialized Nutrition Care.
- A patient can be returned to Standard Nutrition Care if their intake is significantly improved and the current diet prescription meets their needs.

When a patient requires Comprehensive Nutrition Assessment and Specialized Nutrition Care

Comprehensive Nutrition Assessment and Specialized Nutrition Care

- Continue Advanced Nutrition Care procedures, PLUS
- A dietitian should conduct a comprehensive nutrition assessment and undertake active treatment to improve nutritional status. This is specifically recommended for SGA C patients.
- Comprehensive nutrition assessment should occur immediately after SGA rating, or ASAP.
- A comprehensive nutrition assessment may include additional physical nutrition examination, body composition, dietary assessment, clinical history, and biochemical markers, as well as evaluation of swallowing function if required.
- Additional barriers to food intake may need to be investigated (i.e. pain management, nausea, depression).
- Treatment is typically specialized and requires an individualized nutritional care plan.
- Enteral or parenteral nutrition or other treatments that are not provided as part of Advanced Nutrition Care may be required to meet the nutrition needs of patients.
- Collaborate with and educate the patient/family and health care team to help the patient improve their food intake.
- Frequency of follow-up and types of monitoring data collected will be individualized.
- A patient can be returned to Advanced Nutrition Care if their intake is significantly improved and the current diet prescription meets their needs.

At Discharge: All patients receiving Advanced or Specialized Nutrition Care

Post-Discharge Nutrition Care

- Patients who are identified to be malnourished (SGA B or C) and who do not fully recover their nutritional status during their admission, require ongoing care in the community.
- Educate the patient and family on key community resources that can support access to food (e.g. meal programs, grocery shopping services).
- Educate the patient and family on key aspects of their nutrition care plan to support continued recovery in the community.
- Provide details for patients, primary caregivers, and other practitioners involved in post-discharge care, about the patient's nutritional status (e.g. SGA rating, body weight) and treatment provided during hospitalization, as well as recommendations for continuing this care. Request a referral for ongoing nutritional treatment by a dietitian when rehabilitation of nutritional status is on-going.

The Integrated Nutrition Pathway for Acute Care was funded by the Canadian Frailty Network (CFN), which is supported by the Government of Canada through the Networks of Centres of Excellence (NCE).

For more information, and details regarding how to implement INPAC, please visit:
http://www.nutritioncareincanada.ca/inpac/inpac-toolkit
